# Facebook for Scientists: Requirements and Services for Optimizing How Scientific Collaborations Are Established

**DOI:** 10.2196/jmir.1047

**Published:** 2008-08-13

**Authors:** Titus Schleyer, Heiko Spallek, Brian S Butler, Sushmita Subramanian, Daniel Weiss, M Louisa Poythress, Phijarana Rattanathikun, Gregory Mueller

**Affiliations:** ^7^DeepLocalIncPittsburghPAUSA; ^6^Adobe Systems IncorporatedSan JoseCAUSA; ^5^BrulantIncBeachwoodOHUSA; ^4^The MITRE CorporationBedfordMAUSA; ^3^Intel CorporationSanta ClaraCAUSA; ^2^Joseph M Katz Graduate School of BusinessUniversity of PittsburghPittsburghPAUSA; ^1^Center for Dental InformaticsSchool of Dental MedicineUniversity of PittsburghPittsburghPAUSA

**Keywords:** Expertise locating systems, computer supported collaborative work, information systems, collaborators, research, social networks, translational research

## Abstract

**Background:**

As biomedical research projects become increasingly interdisciplinary and complex, collaboration with appropriate individuals, teams, and institutions becomes ever more crucial to project success. While social networks are extremely important in determining how scientific collaborations are formed, social networking technologies have not yet been studied as a tool to help form scientific collaborations. Many currently emerging expertise locating systems include social networking technologies, but it is unclear whether they make the process of finding collaborators more efficient and effective.

**Objective:**

This study was conducted to answer the following questions: (1) Which requirements should systems for finding collaborators in biomedical science fulfill? and (2) Which information technology services can address these requirements?

**Methods:**

The background research phase encompassed a thorough review of the literature, affinity diagramming, contextual inquiry, and semistructured interviews. This phase yielded five themes suggestive of requirements for systems to support the formation of collaborations. In the next phase, the generative phase, we brainstormed and selected design ideas for formal concept validation with end users. Then, three related, well-validated ideas were selected for implementation and evaluation in a prototype.

**Results:**

Five main themes of systems requirements emerged: (1) beyond expertise, successful collaborations require compatibility with respect to personality, work style, productivity, and many other factors (compatibility); (2) finding appropriate collaborators requires the ability to effectively search in domains other than your own using information that is comprehensive and descriptive (communication); (3) social networks are important for finding potential collaborators, assessing their suitability and compatibility, and establishing contact with them (intermediation); (4) information profiles must be complete, correct, up-to-date, and comprehensive and allow fine-grained control over access to information by different audiences (information quality and access); (5) keeping online profiles up-to-date should require little or no effort and be integrated into the scientist’s existing workflow (motivation). Based on the requirements, 16 design ideas underwent formal validation with end users. Of those, three were chosen to be implemented and evaluated in a system prototype, “Digital|Vita”: maintaining, formatting, and semi-automated updating of biographical information; searching for experts; and building and maintaining the social network and managing document flow.

**Conclusions:**

In addition to quantitative and factual information about potential collaborators, social connectedness, personal and professional compatibility, and power differentials also influence whether collaborations are formed. Current systems only partially model these requirements. Services in Digital|Vita combine an existing workflow, maintaining and formatting biographical information, with collaboration-searching functions in a novel way. Several barriers to the adoption of systems such as Digital|Vita exist, such as potential adoption asymmetries between junior and senior researchers and the tension between public and private information. Developers and researchers may consider one or more of the services described in this paper for implementation in their own expertise locating systems.

## Introduction

Social networking technologies have become one of the latest “killer applications” on the Internet, with some sites such as MySpace and Facebook amassing large numbers of users in a very short period of time [1]. While those sites initially focused on younger demographics such as teenagers and college students, they are now encompassing rapidly growing segments of adult and/or professional users. Professionals are beginning to employ such systems for, among other things, extending their professional networks (ie, by learning about colleagues of colleagues), locating experts to solve specific problems, and finding collaborators.

Social networking approaches have the potential to help scientists find appropriate collaborators more quickly and efficiently than is currently the case. Over the past several decades, science has become significantly more collaborative, both generally [2,3], as well as in biomedicine [4]. The increasing frequency with which the terms *interdisciplinarity* and *multidisciplinarity* appear in the literature [5] illustrates this strong trend toward collaboration. As a result, collaboration with the right individuals, teams, and institutions is becoming ever more crucial to project success. New programmatic initiatives such as the Roadmap [6,7] and the Clinical and Translational Science Award (CTSA) [8] programs of the National Institutes of Health (NIH) and the Janelia Farm Research Campus of Howard Hughes Medical Institute [9] in the United States demonstrate that funding agencies and research organizations are not just passively observing this trend but are actively encouraging it.

Currently, most researchers use one of two primary methods to find new collaborators [10]. One approach is to turn to colleagues in their existing social network [10-12]. Colleagues, especially senior ones or those “in the know,” are often able to quickly identify promising candidates for collaboration, to provide input on their potential compatibility and credibility, and to make an introduction. The second method is to search for potential collaborators through published works [10], done most commonly in online databases such as PubMed and Google Scholar. Information from these databases helps the collaboration seeker gauge the potential collaborator’s competence, credibility, and interest, but it provides no support for gaining access. Soliciting collaboration may begin with “cold calling” if no connection through a third person is possible.

A third method for finding collaborators is to use databases of researchers partially or exclusively designed for the purpose. Knowledge management systems of this type, which include “expertise locating systems” [13], “knowledge communities” [14,15], and “communities of practice” [16,17], all provide, to varying degrees, support for finding experts and, by extension, potential collaborators. In the literature, the functions and definitions of these types of systems are not cleanly separated. It appears that expertise locating systems (also called expertise locator systems) as their core function most directly focus on the ability to find individuals knowledgeable in a particular problem/domain.

The computer-supported cooperative work (CSCW) literature contains numerous examples of systems designed to connect people with each other to solve specific problems [13,18-23]. The Expertise Recommender [13] is a recommendation system to help company workers locate persons best qualified to assist with a specific problem. The Zephyr Help Instance [18] and ReachOut [19] are examples of simple lightweight collaborative systems to tap the expertise within a company. Email is sometimes used to exploit weak and latent ties within a professional community [24]. Most of these systems serve to help a person solve a specific problem at a particular point in time. Consequently, one of their most important functions is to help identify the person who is best equipped to assist with solving the problem in a specified time frame.

In this study, we are focusing on the much bigger challenge of establishing the long-term collaborations typical in biomedical science. In this case, not only are researchers looking for the most qualified expert, but they also will most likely enter into a long-term relationship. Evaluating an individual’s promise for such a long-term relationship requires information, engagement, and effort much beyond what is needed for finding an expert for singular (or even episodic) problem solving. A thorough literature search located only one report of a system [21] specifically designed to help scientists meet this challenge.

In contrast to the dearth of reports in the literature, electronic systems purporting to make it easier to help scientists find collaborators abound. Similar to social networking sites such as Facebook, such systems endeavor to help individuals make connections to others that are not likely to be made in an off-line context [25,26]. Among the more established systems is the Community of Science (COS), which provides a “database of detailed, first-person profiles of more than 480,000 R&D professionals and scholars” [27]. Another system, the Faculty Research Interest Project (FRIP) [21], is in use at the University of Pittsburgh and currently indexes 1926 research faculty of the six schools comprising the Health Sciences Center. Another system, ExpertFinder [20], has been designed to help employees of The MITRE Corporation locate experts within the company. LinkedIn, Innocentive, Index Copernicus Scientists, Research Crossroads, and BiomedExperts are some of the more recent commercial offerings that advertise large directories of professionals/scientists. A thorough search for literature evaluating how well these systems facilitate the initiation of collaborations yielded no results. While these systems provide significant value to individuals looking for someone with specific expertise, anecdotal evidence suggests that they currently do not play a significant role in helping researchers establish collaborations.

However, there are good reasons to suspect that expertise locating systems could help scientists find the most appropriate collaborator(s) more quickly and efficiently than is currently the case [26]. General trends in scientific research are compelling scientists to become more collaborative than they already are. As academic/research institutions extend the scale and scope of their research portfolio and, in the process, the numbers of their research faculty, more individuals are available for collaboration, either locally or remotely. At the same time, online databases, such as Google and PubMed, make locating collaborators easier. The number of potential collaborators is also increased by modern communication and collaborative technologies—many remote collaborations that would have once been considered impractical have now become feasible. The result is an “embarrassment of riches” for scientists seeking collaborators. Unfortunately, with this ever-expanding pool of potential collaborators, the task of selecting optimal collaborators is becoming more onerous and requires more effort from researchers, simply because there may be many more good options to choose from than previously possible. Studies have shown that when faced with this type of social overload, individuals are more likely to adopt competitive or withdrawal strategies and thus tend to be less cooperative [28]. Systems that help scientists “quality filter” the realm of possibilities for the most promising potential collaborators could help alleviate this social overload [29] and achieve more appropriate collaboration decisions at lower cost to the collaboration seeker.

The confluence of the trends of increasing scientific collaboration, the emergence of social networking as a powerful mediator of social interaction, and the growing availability of information about scientists and their work presents a significant opportunity to investigate whether expertise locating systems can make the process of finding collaborators more effective and efficient. Current systems are relatively new and have an uncertain track record. One immediate question that occurs is whether those systems are responsive to the requirements of scientists seeking collaborators. In answer to this question, the main goal of this study was to develop preliminary, generalizable requirements for expertise locating systems for biomedical scientists. Its second goal was to design a set of services responsive to these requirements, implement them in a prototype system, and formatively evaluate them with representative end users.

The main focus of this paper is to describe services and functions useful for expertise locating systems in general, not their implementation in a specific system. This study has been conducted as part of the University of Pittsburgh’s Clinical and Translational Science Institute in response to the core challenge to accelerate scientific discovery and the application of its results. As the other 23 current CTSA awardees in the United States are pursuing the same goal, our results are highly significant in that context. In addition, we hope that scientists and developers of expertise locating systems consider our results in the context of their own projects, potentially adopt/implement them, and conceptualize and design additional services as necessary.

## Methods

This project proceeded in two phases: the background research phase and the generative phase. While the background research phase of the project emphasized discovering as much as possible about the relevant problem domain, the generative phase was intended to develop as many viable solutions as possible and then to choose one or more approaches to implement in a prototype. The project team included two faculty from the Center for Dental Informatics (TS and HS), one faculty from the Katz Graduate School of Business (BB) at the University of Pittsburgh, and two faculty (Susan Fussell and Brad Myers) and five senior masters students (SS, DW, LP, PR, and GM) from the Human-Computer Interaction Institute at Carnegie Mellon University. The project took place from January to August 2007. We describe the two main project phases briefly below.

### Background Research Phase

We began the background research phase with a systematic literature review on relevant topics from the computer-mediated communication, social network theory, and computer-supported cooperative work literature. Keywords included “expertise locating systems,” “expertise management systems,” “knowledge communities,” “knowledge management,” “knowledge management systems,” “communities of practice,” and “virtual communities.” We searched Medline, the ISI Web of Science, the ACM Portal, and the IEEE Digital Library (all available years). From this material, we generated an affinity diagram [30] of issues and questions involved in the initiation of collaboration. We then performed contextual inquiries (CI) [31] with 10 researchers at Carnegie Mellon University and the University of Pittsburgh from a range of disciplines and levels of seniority. Since we could not directly observe researchers forming collaborations, the contextual inquiry was based on retrospective accounts. We also used a technique called directed storytelling in which we presented hypothetical situations to the interviewees and had them walk us through what they would do in each given situation. For each CI session, we generated workflow, sequence, and cultural models [31].

**Table 1:** Researcher affiliation, gender, seniority, collaborator count, and perceived collaborative load

School	Gender	Seniority	Number of Collaborators	Perceived Collaborative Load
Medicine	M	Junior	3-4	too few
Medicine	F	Junior	4	too few
Medicine	M	Senior	4	too few
Medicine	F	Junior	7	too few
Dental	M	Junior	7	too few
Rehabilitation	M	Senior	9	too few
Public Health	F	Junior	10	too few
Pharmacy	F	Junior	25	too few
Pharmacy	M	Junior	6	just right
Medicine	M	Senior	6-8	just right
Medicine	F	Junior	8	just right
Nursing	F	Junior	8	just right
Rehabilitation	M	Senior	8	just right
Pharmacy	M	Junior	9	just right
Pharmacy	M	Senior	9	just right
Medicine	M	Junior	10	just right
Medicine	M	Senior	10	just right
Dental	M	Senior	15	just right
Nursing	F	Junior	20	just right
Medicine	F	Junior	20	just right
Public Health	M	Senior	30	just right
Rehabilitation	M	Junior	30-50	just right
Public Health	M	Senior	16-20	too many
Medicine	M	Senior	24	too many
Public Health	F	Senior	40	too many
Public Health	M	Senior	7	n/a
Dental	M	Senior	15	n/a

In a parallel study, we conducted semistructured interviews with 27 scientists at the University of Pittsburgh (see Table 1). The interviews contained 10 main questions and focused on current and previous collaborations, finding collaborators, solving problems in research, and information needs and information resource use of participants. The interview study was conceived as a pilot study since few formal investigations of these topics have been reported in the literature [10]. The interviewers conducted the interviews individually and transcribed their notes shortly thereafter.

We analyzed the semistructured interviews using grounded theory [32], an approach in which the interviewer and one other researcher annotated each transcript independently. Annotations were formulated as themes from which the annotators induced initial hypotheses about the attitudes, motivation, and behavior of the interviewees. A third researcher summarized all annotations and themes, as well as whether they supported or refuted the particular hypothesis or hypotheses they related to.

We modeled three of the semistructured interviews in accordance with the CI method described above and added the resulting workflow, sequence, and cultural models to the 10 sets of models developed during the CI phase. We did this in order to increase the variety of observations and add insights that may have been articulated during the interviews but not during the CI sessions. Subsequently, we consolidated the data into single flow, sequence, and cultural models. The flow model provided a good view of actors and their roles and the flow of information among them. The cultural model identified the cultural aspects that have a strong influence on whether and how collaborations are formed.

We then derived a detailed set of requirements from the consolidated models and the results of the interviews and categorized them into five main themes: compatibility, communication, intermediation, information quality and access, and motivation. These themes served as the basis for developing the design ideas during the generative phase, which we describe next.

### Generative Phase

The generative phase began with brainstorming design ideas for systems to help facilitate the establishment of collaborations in light of the system requirements we had formulated. Two initial brainstorming sessions resulted in a total of over 40 ideas. The ideas included semiautomatic updating of online profiles; locating collaborators through colleagues or matching research interests in published papers; utilizing online journal clubs, online video presentations, and live question-and-answer sessions; social tagging of research papers; facilitating directed social contact through methods such as ride sharing and hobby groups; and creating systems to support matchmaking through “social hubs,” such as department chairs. Several of the ideas drew on functions available in the Web 2.0 and ubiquitous/mobile computing technology spaces.

Sixteen of the 40 ideas generated during the brainstorming phase were selected for formal concept validation. During this phase, we evaluated the design concepts with nine researchers at the University of Pittsburgh Health Science Campus. The participants represented scientists at the junior, senior, and executive levels with varying research foci (basic, clinical, and translational) at several schools. We presented each design idea as a real-life scenario to the participant and solicited feedback on its functionality and usefulness. Thus, we used the viewpoint of the end user as a central guiding principle for shaping our designs, an approach crucial to the development of user-centered applications [31]. The scenarios employed “personae,” which are archetypal representations of individuals that represent either the participant or individuals they would encounter when interacting with the system. For instance, “Carlos” was characterized as an inexperienced junior researcher at the School of Pharmacy in the early stages of his career. He had few contacts and was willing to be less selective about collaborative projects in order to gain experience and expand his network. “Bernice,” on the other hand, was a well-known biomedical researcher who demanded a rigorous work style and could afford to discontinue collaborations she felt were fruitless. The personae brought life to the scenarios and allowed participants to act and react naturally with regard to the proposed ideas. A facilitator presented the scenarios and guided user feedback through scenario-specific questions. At least one other observer was present to record notes. The sessions were audio-recorded as a reference for analysis.

For each design idea, the individual ratings of the researchers were combined into a summary score that ranged from 1 (not needed) to 4 (very much needed). At the same time, the project team rated the feasibility of implementing each idea on a scale from 1 (low) to 3 (high). The feasibility rating integrated judgments about how difficult it would be to implement each idea based on technical, environmental, and cultural considerations.

Based on the feedback from the concept validation sessions, we selected three related ideas for implementation and evaluation in a prototype. We implemented the design first as a wire frame, then as a high fidelity prototype. We performed think-aloud evaluations with four scientists using three use cases. The use cases described common scenarios that we asked participants to complete using the Digital|Vita prototype. Two observers kept notes on the interaction of each participant with the system, focusing on functions that were found to be either problematic or useful. The development team then brainstormed system improvements and implemented them to the highest degree possible. The high-fidelity prototype was used to produce a video about the system, which served as a way to solicit input from senior decision makers and external reviewers.

The studies conducted as part of the background research phase and generative phase were approved by the University of Pittsburgh Institutional Review Board (IRB approval numbers: 0612065 and PRO07050299).

## Results

The background literature review, affinity diagramming, contextual inquiry, and semistructured interviews yielded five themes of requirements for systems to support the formation of collaborations. The themes are compatibility, communication, intermediation, information quality and access, and motivation. We briefly describe the themes below.

### Themes for System Requirements

#### Compatibility

Beyond expertise, successful collaborations require compatibility with respect to personality, work style, productivity, and many other factors [10,33]. Although exceptions exist, the majority of researchers interviewed saw compatibility of personality and work style as a prerequisite to collaboration. Therefore, more than a simple overlap of interests is needed to create a successful collaboration. The researchers we interviewed indicated that they would not trust an impersonal recommendation or suggestion made by a system about potential compatibility, putting them somewhat at odds with what users of dating sites and Facebook are apparently willing to do [34,35]. If researchers cannot assess compatibility with potential collaborators personally, they primarily appear to trust personal recommendations from colleagues. For this reason, expertise locating systems should show social connections between the collaboration seeker and potential collaborators.

#### Communication

Finding appropriate collaborators requires the ability to effectively search in domains other than your own using information that is comprehensive and descriptive [29,36]. However, researchers are often unlikely to be very familiar with the terminology they need in order to find a specific area of expertise in another domain [29]. One way that researchers currently solve this problem is by asking boundary-spanning colleagues and friends familiar with both realms about whom they should contact for help with solving a particular research problem. While the system should provide researchers with the ability to search directly for expertise, it should also make explicit who in their own professional network may be able to guide them effectively to other experts for resolving questions in different disciplines, organizational units, or research groups. A second requirement for communication is to broaden the ability to search for experts using more information than just publication databases such as Medline or Google Scholar. Those databases typically describe the knowledge and expertise of a researcher in less detail and less comprehensively than a complete curriculum vitae (CV). The CV can support a richer form of evaluation because it provides a more complete picture of the individual’s research-related activities, such as grants, grant reviews, patents, editorships, and positions in associations. Expertise locating systems should therefore not only allow the user to search a potential collaborator’s publications, but also their research interests, grant submissions, and biographical information.

#### Intermediation

Social networks are very important for finding potential collaborators, assessing their suitability and compatibility, and establishing contact with them. Established researchers often use existing connections with colleagues as their primary resource for locating new collaborators. However, junior researchers with few or no contacts within the desired field may have significant difficulty initiating collaborations [26]. Researchers are more likely to contact a friend or colleague who they think will know an expert than to cold-call the author of a relevant research paper [24]. Advantages of personal contact include a higher likelihood of compatibility between parties, increased chances of a timely response (which is an issue when there is a status differential), and a less intimidating (and potentially face-saving) method of contacting a new party. Websites such as Facebook and LinkedIn circumvent cold-calling by integrating recommendation services and allowing users to see friends of friends. In this way, users’ networks are actually expanded to include their friends’ networks in addition to their own. The tendency to use friends/colleagues as intermediaries strongly supports the power and influence of existing social networks and suggests that a successful collaboration-networking site will need to leverage this construct for both identification and access [11,12]. Eysenbach [Eysenbach Medicine 2.0 Editorial, this issue] suggests the new scholarly term “apomediation” for the function of the intermediary, emphasizing the positive guidance toward high-quality resources (in this case, collaborators).

#### Information Quality and Access

Information profiles must be complete, correct, up-to-date, and comprehensive and allow fine-grained control over access to information by different audiences. Missing, incorrect, and out-of-date information and poor indexing (for instance, through the use of nonstandard vocabularies) of information profiles [15,20,29,37] make it difficult for a collaboration seeker to obtain the information necessary to assess the suitability and appropriateness of a prospective collaborator. Several commercial services, such as the Community of Science, rely on the user to keep their profile up-to-date and correct at all times. Others generate a “preliminary” profile for scientists from public sources, such as PubMed, Computer Retrieval of Information on Scientific Projects (CRISP), and the USPTO database, which the individual must correct and complete in order to generate a comprehensive, up-to-date profile. Several researchers we interviewed indicated that they had public online profiles but that they did not spend much time keeping them current. Given the many factors that collaboration seekers take into account when evaluating potential collaborations, information profiles should be as complete, correct, and up-to-date as possible [26]. In addition, some researchers indicated that given a choice, they would be selective about what information they would consider making public about themselves. For instance, interviewees seemed much more willing to disclose current ideas to collaborators from within their institutions than to those from competing institutions.

#### Motivation

Keeping online profiles up-to-date should require little or no effort and be integrated into the scientist’s existing workflow. In order to provide relevant and up-to-date information to colleagues, researchers must have an incentive to supply the information and keep it current. For example, our study participants regularly invested time updating information in their biographical and professional documents, such as their CV, biosketches for grants, and faculty evaluation forms. They were highly motivated to do so because these documents must be up-to-date in order to obtain grant funding, provide background information when invited to lecture or consult, and participate in university evaluations such as promotion and/or tenure decisions. There is no such motivation to update online profiles. In addition, our contextual inquiries showed that a major problem with the existing workflow is that researchers need to reformat and update the same information in multiple documents. We recognized this as an opportunity to draw researchers into using an online system. If it were possible to streamline the process by synchronizing information in multiple documents, the system would provide an incentive to keep information updated. Therefore, before a database of personal researcher profiles can be used as a tool to initiate collaboration, it must to be adopted as a repository of biographical and research-related information by a wide range of researchers. This “critical mass” problem is one of the classic challenges to the adoption of CSCW systems [38].

**Figure 1:**
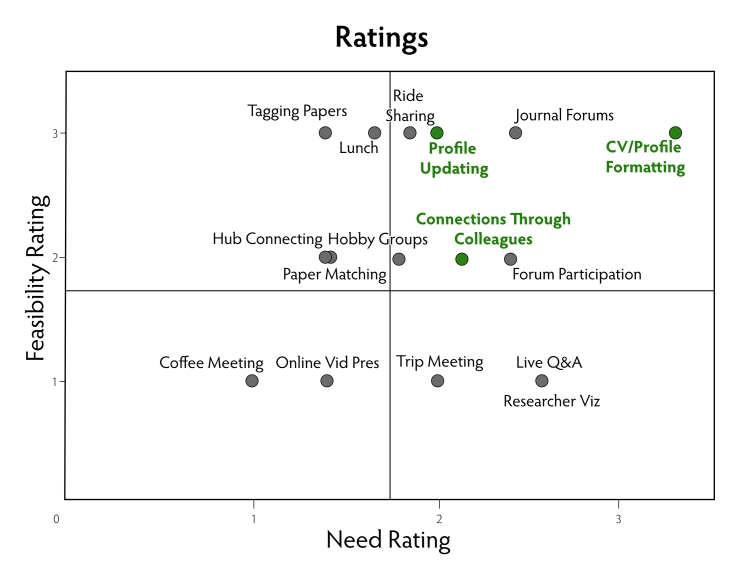
Results of the concept validation phase for 16 design ideas using nine scientist participants; need rating: 1 (low) to 4 (high); feasibility rating: 1 (low) to 3 (high)

### Services for Optimizing How Scientific Collaborations Are Established

As described in the Methods section, the research team generated a large number of ideas for one or more systems to support researchers in locating collaborators. Figure 1 shows the results of the concept validation phase for the 16 design ideas. It is important to note that there is not a 1:1 correspondence between the requirement themes and application ideas. Rather, the different application ideas are responsive to one or more requirement themes to different degrees. In selecting the ideas to be implemented, we aimed to respond to the requirements as best as possible within the context of a software application. The combination of three highly validated ideas, Profile Updating, CV/Profile Formatting, and Connections through Colleagues, appeared to satisfy our constraints most closely and were chosen to be implemented in a prototype system which we dubbed “Digital|Vita.” The three main sets of services implemented in Digital|Vita are the following:

1. Maintaining, formatting, and semiautomated updating of biographical information: This set of services allows users to maintain biographical information and output it to several standard formats.

2. Searching for experts: These services provide capabilities for searching for potential collaborators using a range of search criteria and allow searchers to exploit the social network represented in Digital|Vita in the process.

3. Building and maintaining the social network and managing document flow: These services allow users to build a network of social connections, group colleagues into teams, and manage the flow of biographical documents within their teams.

The following sections briefly describe these sets of services.

#### Maintaining, Formatting, and Semiautomated Updating of Biographical Information

**Figure 2:**
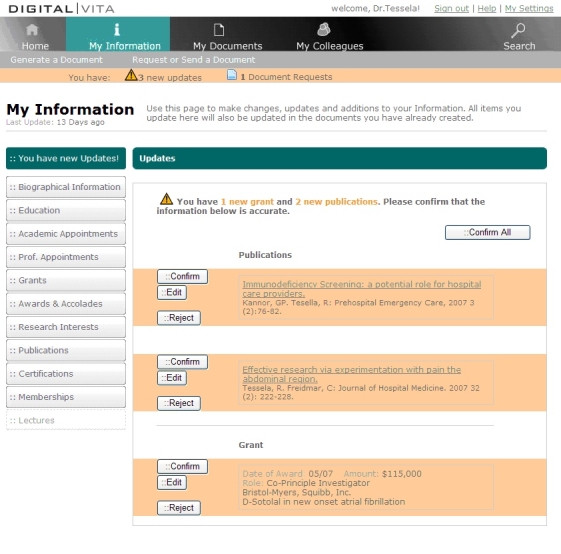
The My Information component in Digital|Vita allows the user to enter and update biographical information through manual or semiautomated processes

This service is provided by the My Information (see Figure 2) data management function in DigitalVita, which stores biographical information about a user in a comprehensive and detailed manner. Information typically found in CVs, such as education, academic appointments, grants, and publications, can be entered and edited by the user. The items making up each collection, such as single publications, are stored as separate records and logically divided into fields in the database, enabling fine-grained information extraction and display.

Three services in My Information allow the user to enter and update biographical information:

1. Importing information from existing sources: The primary method for populating biographical information is extraction from existing sources such as the National Library of Medicine’s Medline and the National Institutes of Health’s CRISP databases. A similar approach to retrieving and aggregating data from existing sources is being used in many other systems [13,20-23]. In Digital|Vita, records from these sources are pre-matched (for instance, through a name search) to the user, and the user simply confirms which records pertain to them. (This approach is used by the Faculty Research Interests Project (FRIP) [21] system currently in use at the University of Pittsburgh. When Digital|Vita is implemented at the University of Pittsburgh, publications will be imported from the existing FRIP database.)
2. Propagating information through social networks: A second mechanism for acquiring biographical information is the semiautomated synchronization of updates made by colleagues in Digital|Vita. The process is semiautomated because all affected scientists are automatically notified about updated information, but each of them has to manually approve the update for inclusion in their own information. For instance, when Digital|Vita users manually enter a paper, they have the option of selecting coauthors from within the Digital|Vita system. When an entry is saved, Digital|Vita automatically propagates this update to the coauthors and displays it on each user’s Digital|Vita home page (see Figure 2). The coauthors can then confirm or reject the update for their own personal profile.
3. Manually entering and updating information: The third mechanism for entering and updating information is manual entry. This is appropriate for data for which existing sources are neither available nor accessible. Examples of such data include professional appointments, degrees, and publications indexed in services that do not allow automated retrieval.

**Figure 3:**
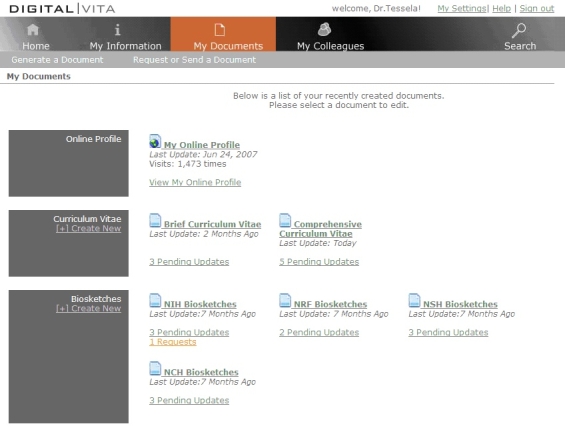
The My Documents component provides functions to output biographical information to several standard formats, customize information content, archive old versions, and include updates to biographical information selectively

While My Information allows the user to input and manage their biographical information, the My Documents function helps the user produce and archive several forms of output from that data. My Documents includes three services:

1. Output to several standard formats: The current design of Digital|Vita provides for several standard output formats for biographical information such as a university-specific CV, a brief CV, and NIH and NSF biosketches. Users can choose the desired output format, generate the new document, and edit it according to their preferences.
2. Customization and versioning: The ability to easily customize document content was deemed essential for the researchers we interviewed because they typically adapt biographical documents for specific grant applications, even if the format required for each is the same. With this service, users can customize documents with a simple checkbox approach—if an item is checked, it is included in the specific document. My Documents also supports versioning so that older versions of a specific document are available on demand.
3. Selective updating: The system makes it explicit when the existing version of a document does not include recently updated information (see Figure 3) in order to allow the user to make an informed choice about including or excluding such updates. When the user customizes documents with information that is not contained in the My Information database, Digital|Vita allows the user to back-propagate the information to My Information. Thus, users do not have to interrupt their current workflow in order to make updates to My Information.

As the user edits a specific document, the system displays the length of the document in pages in order to allow the user to observe page limits. In addition, the user can preview the printed version of the document; send it to colleagues in their professional network and recipients through email; and save the document in predetermined file formats.

#### Searching for Experts

Our background research indicated that researchers consider a variety of factors when choosing potential collaborators. For many, searching Medline and Google Scholar is only the first step in acquiring several types of information about their colleagues. The purpose of the My Information section in Digital|Vita is to store rich and comprehensive profiles of researchers in the database and make them available for flexible and powerful searching by others.

**Figure 4:**
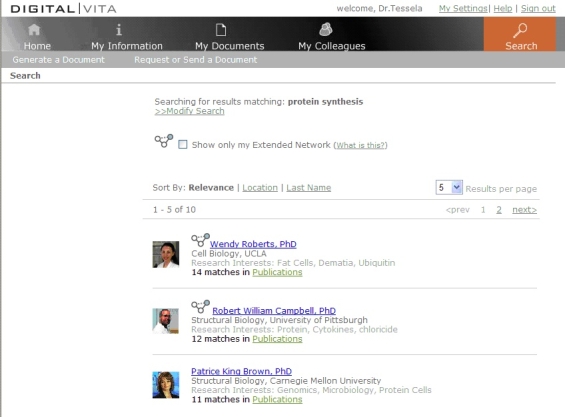
A sample search results screen in Digital|Vita shows brief profiles of potential collaborators

1. Simple and advanced search of profiles: The first step in finding an expert within Digital|Vita is to allow users to query profiles flexibly. While the simple search in Digital|Vita only offers the capability to query profiles using keywords, the advanced search adds institution, department, location (for institutions with multiple campuses), publication activity, and relevance. (Relevance is a score indicating the level of expertise of the “hit” regarding the desired research topic.) Search results return key information about each hit (see Figure 4). They include academic affiliation, research interests, publications, and number of citations. Users can sort the search results and compare the appropriateness of potential collaborators. A potential trade-off of this design results from the fact that status, seniority, and relative experience of a person are now explicitly communicated. This could affect the decisions collaboration seekers make because a well-published and experienced researcher is now clearly identifiable as compared to a less published, less experienced researcher. Making these distinctions highly visible may potentially reduce the opportunities junior researchers are offered. On the other hand, it may allow the searcher to target a collaborator’s level of experience and expertise more directly. When users have identified one or more promising candidates for collaboration, they can access detailed profiles. Researchers’ profile pages contain information they have approved for inclusion by managing the My Profile section of their Digital|Vita. Thus, researchers have relatively granular control over which information is published about them. Typically, the profile page displays detailed information about their background, research interests, and publications (with links to PubMed for abstracts and, in some cases, full-text articles).
2. Exploiting the social network to search: The search results page also displays connections through colleagues (identified by an icon symbolizing a social network, see icon next to "Wendy Roberts" in Figure 4). The icon shows two nodes if the individual is a colleague who belongs to the user’s social network and three nodes if the individual is a colleague of a colleague. This design paradigm resembles the functionality of sites such as Facebook and LinkedIn. Users may elect to search only in their extended network (ie, among individuals who are in the social network of their colleagues). Junior researchers may find this feature helpful to avoid having to cold-call potential collaborators. The Digital|Vita design currently does not provide a mechanism for asking a colleague for an introduction electronically, as other systems do.

#### Building and Maintaining the Social Network and Managing Document Flow

This service is managed in the My Colleagues section of Digital|Vita. It is intended for researchers to keep track of their collaborators, colleagues within their department, and general professional network of colleagues within Digital|Vita. It is the area in the system where users build the social network that they are able to exploit when they search for collaborators (see above) and where they manage document flow between themselves and their research teams.

1. Creating links to colleagues: The value of social networks for recommending collaborators has been discussed earlier [39-41]. An obvious hurdle to establishing a social network is that there are few information sources from which data can be drawn to populate it directly. Nonetheless, in order to reduce the work for users, Digital|Vita generates suggestions for individuals to be included in a user’s social network by matching coauthors on papers and collaborators on grants with existing researchers in Digital|Vita. Users can then decide individually whether to include the suggested individuals in their social network. However, coauthorship and collaboration on grants are typically no more than partial indicators of collaboration [3]. Therefore, Digital|Vita users can ask anyone in the system to become their colleague. In this case, the system sends an electronic invitation, which the recipient either can accept or reject. In case of rejection, the recipient can opt to provide a reason. The requester is then notified about the recipient’s decision. One design alternative that was considered was not to notify the requester about the recipient’s decision. This alternative would provide a way to save face for both parties [42]. We decided against this design, however, in order to prevent users from thinking that the system was not working when receiving no response to their request.
2. Assembling research teams: My Colleagues also provides a simple mechanism to label and organize groups of colleagues. The primary use of these groups is to manage the flow of biographical documents among them. The rationale for this feature is that many collaborations in academia arise within the context of pursuing a particular funding opportunity. Since one key activity in preparing grant applications is collecting biographical information from each team member, it was logical to add functions to Digital|Vita to support this effort. Users can create any number of named groups drawing from their list of colleagues on record in the system. As in real life, individual colleagues can belong to more than one group. Groups can be annotated with relevant information, for instance with the identifier of the funding opportunity the group is working on.
3. Managing biographical document flow: Once a Digital|Vita user has created a named group, he or she can issue an electronic request to the group specifying the type of document requested (eg, NIH biosketch), the purpose for the request, and the date the information is needed by. Team members respond to requests through the system, which gives each person the opportunity to customize the requested document before it is sent. Digital|Vita issues automatic reminders to team members who have not responded by the due date. (Requesting documents in this manner is also possible between individuals.) The status of requests to and from other colleagues, as well as responses, is tracked in a Document Inbox. The Document Inbox allows users to send or request a document, as well as view and manage their recent document requests. Historical requests are accessible through a link to an archive. Before sending a document, users can preview it to ensure they are sending the correct document and that it contains the desired information. If new additions to the user’s biographical information have been made, the document can be edited directly before sending. Users can also decline a document request. Requests are archived automatically after the due date of the document has passed or when the user has sent the requested document.

In summary, maintaining, formatting, and semiautomated updating of biographical information; searching for experts; and building and maintaining the social network and managing document flow are three sets of services designed to make the process of finding collaborators more efficient and effective and so facilitate the establishment of collaborations. We have focused on describing the Digital|Vita functionality as separate services in order to allow other researchers and developers to implement them selectively or all together in other systems.

### Additional Information About Digital|Vita

The preceding section presents a relatively abbreviated description of the functionality of the Digital|Vita system. A video illustrating a prototype of the system and its use is available in the Multimedia Appendix. In addition, the final report (dated July 2007) about the Digital|Vita prototype project, which includes a comprehensive description of the problem space, research, and development methods and the Digital|Vita design and functionality, including the design rationale, is available online. At present, the Digital|Vita development team is writing detailed system specifications for the development of a production application.

## Discussion

The problem of connecting scientists with each other is not new. However, doing so efficiently and effectively has taken on particular relevance and urgency in an age when much of science is migrating to a multidisciplinary, collaborative, and team-oriented model. At the same time, while electronic systems to help connect scientists have existed for some time, to this point they appear to have played only a minor role in helping scientists form collaborations.

Systematic approaches to designing systems to help researchers find collaborators are only in their infancy. We began this study with two basic research questions: (1) What requirements should systems for finding collaborators in biomedical science fulfill? and (2) Which information technology services can address these requirements? We believe that we have made an important contribution to the design of expertise locating systems with regard to both questions. The five main themes we have identified as requirements for such systems (compatibility, communication, intermediation, information quality and access, and motivation) show that collaboration seeking is a complex activity that does not depend simply on the ability to retrieve factual information about potential collaborators. It is clear from our exploration of these themes that social connectedness, personal and professional compatibility, and power differentials influence the formation of collaborations. This means that systems that do not model and leverage the social context are at a clear disadvantage in satisfying the social requirements for establishing collaborations.

On the other hand, a rich informational representation of potential collaborators also appears to be important. Checking PubMed and Google for publications of a potential collaborator was only a starting point for many of our interviewees. Detailed investigation included other information resources, such as the NIH’s CRISP, as well as patent and other databases. Because of the fragmentation of information about potential candidates, a thorough background search on potential collaborators is time and effort intensive. The cost of a search, therefore, appeared to be a barrier to finding the most appropriate and qualified collaborators. It was therefore logical for our design to focus on the most comprehensive and up-to-date, but customarily also least accessible, information profile available: the CV of the individual scientist.

Systems such as the Community of Science have long made biographical information a centerpiece of researcher profiles. Why do we think Digital|Vita may succeed where others have failed? Digital|Vita is centered on one component of a workflow that scientists almost universally perform on an ongoing basis—maintaining and updating the CV—and adds functions to support the establishment of collaborations. This design mirrors Payton’s [37] approach to use trails through an information space to identify individuals with common interests. In both cases, information useful for expertise location is a by-product of activities that are already being performed. In addition, CV maintenance in Digital|Vita remains in its local context. Moreover, institutions typically have idiosyncratic formats for CVs and evaluations, and thus systems designed to manage biographical information must be able to format it according to local requirements. To our knowledge, while DigitalVita includes this function, none of the major commercial expertise locating systems, such as the CoS, Collexis, and Research Crossroads, provide this functionality, which is a major barrier to their adoption.

Managing biographical information within Digital|Vita not only requires no extra effort from a scientist compared to the traditional approach, it actually reduces effort because the raw biographical information is converted automatically to several frequently used standard formats. Making this workflow a central feature of Digital|Vita may prompt researchers to at least explore the collaboration-seeking functionality of the system.

However, the simple availability of features to search for collaborators does not mean that they will be used. Encouraging researchers to seek collaborators through Digital|Vita as opposed to traditional methods faces significant obstacles. For instance, established researchers often are so well-informed and well-connected that they, on average, will outperform any electronic system. We therefore anticipate that Digital|Vita may be primarily attractive to younger scientists (who may be using social networking tools in their life outside of work) and scientists who are new to the University of Pittsburgh or who are planning to collaborate with individuals in disciplines that they are not very familiar with. Digital|Vita also faces a complex challenge in keeping information about a researcher private while at the same time marketing that researcher to maximum effect. We believe that the granular control Digital|Vita provides in determining what information is public and what is not will help individuals adjust their public profile to their preferences. Other potential barriers to adoption include establishing an initial critical mass of profiles adequate for finding and choosing collaborators and integrating the systems and its capabilities with the regular work practice of the institution and individual researchers.

Future work on the Digital|Vita system will take two major directions. After development and implementation of the production system, we plan to design additional functions intended to improve the matching process among potential collaborators. Most likely, this research strand will focus on the development of algorithms to help pinpoint the most promising collaborators and bring new potential collaborative opportunities to a researcher’s attention. A second direction for the Digital|Vita effort will be to identify other information technology services to help scientists find and access resources that are useful for their work. For instance, we are currently working on a directory of computational resources at the University of Pittsburgh to support scientific problem solving.

It is clear that electronic systems in support of research, and specifically those supporting the establishment of collaborations, will become increasingly important in the future. As more and more science goes “digital,” both in its execution as well as in its documentation, systems such as Digital|Vita will become essential to the everyday life and activities of scientists.

## Multimedia Appendix

Prototype of the Digital|Vita system (Video)

## Abbreviations

CI: contextual inquiry
CRISP: Computer Retrieval of Information on Scientific Projects
CV: curriculum vitae
NIH: National Institutes of Health
